# The sarcoma ring trial: a case-based analysis of inter-center agreement across 21 German-speaking sarcoma centers

**DOI:** 10.1007/s00432-024-06063-z

**Published:** 2025-01-04

**Authors:** Siyer Roohani, Jolina Handtke, Kamal Hummedah, Markus Albertsmeier, Dimosthenis Andreou, Leonidas Apostolidis, Marinela Augustin, Sebastian Bauer, Moritz Billner, Florian Bösch, Christoph K. W. Deinzer, Niklas Deventer, Anna Duprée, Franziska Eckert, Lars Engel, Katja Fechner, Hagen Fritzsche, Verena Gaidzik, Saeed Ghani, Robert Grützmann, Wiebke K. Guder, Rainer Hamacher, Judith S. Hecker, Anne Hendricks, Axel Hillmann, Philipp Houben, Georg Hübner, Philipp Ivanyi, Christina Jentsch, Maren Jordan, Peter Kappl, Moritz Kaths, Torsten Kessler, Johanna Kirchberg, Carolin Knebel, Robert Krempien, Burkhard Lehner, Ulrich Lenze, Lars H. Lindner, Alisa Martina Lörsch, Nadia Maguire, Sophie Müller, Pompiliu Piso, Vlatko Potkrajcic, Peter Reichardt, Stephan Richter, Simone Schewe, Lars M. Schiffmann, Felicitas Scholten, Jana Käthe Striefler, Matthias Schwarzbach, Katharina Seidensaal, Sabine Semrau, Joanna Szkandera, Christoph J. Szuszies, Beate Timmermann, Armin Tuchscherer, Armin Wiegering, Moritz T. Winkelmann, David Kaul, Jens Jakob

**Affiliations:** 1 Department of Radiation Oncology, Charité-Universitätsmedizin Berlin, Corporate Member of Freie Universität Berlin and Humboldt-Universität zu Berlin, Berlin, Germany; 2 Berlin Institute of Health at Charité-Universitätsmedizin Berlin, BIH Biomedical Innovation Academy, BIH Charité (Junior) Clinician Scientist Program, Berlin, Germany; 3 German Cancer Consortium (DKTK), Partner Site Berlin, a Partnership Between DKFZ and Charité-Universitätsmedizin Berlin, Berlin, Germany; 4 Sarcoma Unit, Department of Surgery, University Medical Center and Medical Faculty Mannheim, University of Heidelberg, Mannheim, Germany; 5 DKFZ Hector Cancer Institute at the University Medical Center Mannheim, Mannheim, Germany; 6 Department of General, Visceral and Transplantation Surgery, Ludwig-Maximilians-Universität (LMU) Munich, LMU University Hospital, Munich, Germany; 7 Department of Orthopedics and Trauma, Medical University of Graz, Graz, Austria; 8 Department of Orthopedic Oncology, West German Cancer Center, University Hospital Essen, University Duisburg-Essen, Essen, Germany; 9 Department of Medical Oncology, Heidelberg University Hospital, National Center for Tumor Diseases (NCT) Heidelberg, Heidelberg, Germany; 10 Department of Hematology and Oncology, Paracelsus Medical University, Nuremberg, Germany; 11 Department of Medical Oncology and Sarcoma Center, West German Cancer Center, University Duisburg-Essen, Medical School, Essen, Germany; 12 DKTK Partner Site Essen, German Cancer Consortium (DKTK), Heidelberg, Germany; 13 Department of Plastic, Reconstructive and Hand Surgery, Center for Severe Burn Injuries, Paracelsus Medical University, Klinikum Nürnberg, Nuremberg, Germany; 14 Department of General, Visceral and Pediatric Surgery, University Medical Center Göttingen, Göttingen, Germany; 15 Department of Internal Medicine VIII-Medical Oncology and Pneumology, University Hospital Tübingen, Tübingen, Germany; 16 Department for General Orthopaedics and Tumor Orthopaedics, University Hospital Muenster, Muenster, Germany; 17 Department of General, Visceral and Thoracic Surgery, University Medical Center Hamburg-Eppendorf, Hamburg, Germany; 18 Department of Radiation Oncology, University Hospital Tübingen, Tübingen, Germany; 19 Department of Radiation Oncology, AKH, Comprehensive Cancer Center Vienna, Medical University Vienna, Vienna, Austria; 20 Department of Visceral-Thoracic and General Surgery, Paracelsus Medical University, Nuremberg, Germany; 21 Department of Surgery, University Hospital, Erlangen, Germany; 22 University Center for Orthopedics, Trauma and Plastic Surgery, Faculty of Medicine and University Hospital Carl Gustav Carus, TUD, Dresden University of Technology, Dresden, Germany; 23 Clinic for Internal Medicine III, University Hospital Ulm, Ulm, Germany; 24 Helios Klinikum Berlin Buch, Sarkomzentrum, Berlin, Germany; 25 Department of General and Visceral Surgery, Friedrich-Alexander-University, Erlangen, Germany; 26 Department of Medicine III, School of Medicine and Health, Technical University of Munich (TUM), Munich, Germany; 27 Department of General, Visceral, Transplantation, Vascular, and Pediatric Surgery and Comprehensive Cancer Center Mainfranken Würzburg, University Hospital Würzburg, Würzburg, Germany; 28 Department of Orthopedic and Trauma Surgery, Barmherzige Brüder Regensburg Medical Center, Regensburg, Germany; 29 Department for General-, Visceral- and Transplant Surgery, University Hospital Muenster, Muenster, Germany; 30 Department of Radiation Oncology, Barmherzige Brüder Regensburg Medical Center, Regensburg, Germany; 31 Clinic for Hematology, Hemostaseology, Oncology and Stem Cell Transplantation, Hannover Medical School, Hannover, Germany; 32 Department of Radiotherapy and Radiation Oncology, Faculty of Medicine and University Hospital Carl Gustav Carus, TUD, Dresden University of Technology, Dresden, Germany; 33 OncoRay-National Center for Radiation Research in Oncology, Dresden, Germany; 34 Nationales Centrum Für Tumorerkrankungen Dresden (NCT/UCC): Deutsches Krebsforschungszentrum (DKFZ), Universitätsklinikum Carl Gustav Carus Dresden, Medizinische Fakultät der Technischen Universität Dresden, Helmholtz-Zentrum Dresden-Rossendorf (HZDR), Dresden, Germany; 35 Varisano Klinikum Frankfurt Höchst, Frankfurt, Germany; 36 Department of Radiology, Neuroradiology, and Nuclear Medicine, Barmherzige Brüder Regensburg Medical Center, Regensburg, Germany; 37 Department of General, Visceral and Transplantation Surgery, Sarcoma Center, West German Cancer Center, University Hospital Essen, University Duisburg-Essen, Essen, Germany; 38 Department of Hematology and Oncology, University Hospital Muenster, Muenster, Germany; 39 Department of General, Visceral, Thoracic and Vascular Surgery, Faculty of Medicine and University Hospital Carl Gustav Carus, TUD Dresden University of Technology, Dresden, Germany; 40 Department of Orthopaedics and Sports Orthopaedic, Klinikum Rechts Der Isar, Technical University of Munich (TUM), Munich, Germany; 41 Clinic for Radiotherapy, HELIOS Klinikum Berlin-Buch, Schwanebecker, Berlin, Germany; 42 MSB Medical School Berlin, Fakultät für Medizin, Berlin, Germany; 43 Department of Orthopaedics, Heidelberg University Hospital, Heidelberg, Germany; 44 SarKUM, Center of Bone and Soft Tissue Tumors, LMU University Hospital, LMU Munich, 81377 Munich, Germany; 45 Department of Medicine III, LMU University Hospital, LMU Munich, 81377 Munich, Germany; 46 Department of Oncology and Hematology, Krankenhaus Barmherzige Brüder Regensburg, Regensburg, Germany; 47 Department of General and Visceral Surgery, Hospital Barmherzige Brüder, Regensburg, Germany; 48 Department of Medicine 1, National Center for Tumor Diseases Dresden (NCT/UCC), Sarcoma Center, University Hospital Carl Gustav Carus Dresden, Dresden University of Technology, Dresden, Germany; 49 Department of General, Visceral, Cancer and Transplant Surgery, Faculty of Medicine and University Hospital of Cologne, University of Cologne, Cologne, Germany; 50 Department of Internal Medicine II, Oncology/Hematology/BMT/Pneumology, University Medical Center Hamburg-Eppendorf, Hamburg, Germany; 51 Department of Radiation Oncology, Heidelberg University Hospital, Heidelberg, Germany; 52 Heidelberg Ion-Beam Therapy Center (HIT), Heidelberg University Hospital, Heidelberg, Germany; 53 National Center for Tumor Diseases (NCT), Heidelberg, Germany; 54 Department of Radiation Oncology, University Hospital Erlangen, Erlangen, Germany; 55 Division of Clinical Oncology, Department of Internal Medicine, Medical University of Graz, Graz, Austria; 56 Department of Hematology and Medical Oncology, University Medical Center Göttingen, Göttingen, Germany; 57 Department of Particle Therapy, University Hospital Essen, West German Proton Therapy Centre Essen (WPE), Essen, Germany; 58 Department I of Internal Medicine, Faculty of Medicine and University Hospital, University of Cologne, Cologne, Germany; 59 Faculty of Medicine and University Hospital, University of Cologne, Center for Integrated Oncology Cologne Aachen Bonn Cologne Duesseldorf (ABCD), Cologne, Germany; 60 Department of Biochemistry and Molecular Biology, University of Würzburg, Würzburg, Germany; 61 Department for Diagnostic and Interventional Radiology, University Hospital Tübingen, Tübingen, Germany; 62 Health and Medical University Potsdam, Potsdam, Germany

**Keywords:** Soft tissue sarcoma, Ring trial, Reference center, Inter-center agreement, German Cancer Society, Guidelines

## Abstract

**Purpose:**

The management of soft tissue sarcoma (STS) at reference centers with specialized multidisciplinary tumor boards (MTB) improves patient survival. The German Cancer Society (DKG) certifies sarcoma centers in German-speaking countries, promoting high standards of care. This study investigated the variability in treatment recommendations for localized STS across different German-speaking tertiary sarcoma centers.

**Methods:**

In this cross-sectional case-based survey study, 5 anonymized patient cases with imaging data of localized STS were presented to MTBs of 21 German-speaking tertiary referral hospitals. Centers provided recommendations on treatment sequence and modalities, along with the consensus level within their MTB. Agreement percentages were calculated, and consensus levels were rated on a scale of 1 to 10.

**Results:**

Five patient cases were discussed resulting in 105 recommendations. Agreement percentages for case 1 to 5 were 14.3%, 61.9%, 33.3%, 52.4% and 9.3%, with a median agreement percentage of 33.3%. Grouping pre- and postoperative therapies as "perioperative" and including recommendations with and without regional hyperthermia raised the median agreement to 47.6%. The mean consensus level within each center across all 5 cases was 9.5.

**Conclusion:**

This first case-based analysis of inter-center agreement for STS management in German-speaking countries reveals low inter-center agreement but high intra-center consensus. Our study includes nearly all tertiary sarcoma centers in German-speaking countries, affirming its strong external validity. These findings suggest potential and clinically very relevant differences in treatment standards among sarcoma centers. Enhanced case-based exchanges and collaborative efforts are needed to reduce discrepancies and standardize the management of STS patients.

**Supplementary Information:**

The online version contains supplementary material available at 10.1007/s00432-024-06063-z.

## Introduction

The management of soft tissue sarcoma (STS) at reference centers with specialized multidisciplinary tumor boards (MTB) improves patient survival (Bonvalot et al. 2019; Blay et al. 2019; Tirotta et al. 2023a). International and country-specific guidelines, as well as sarcoma expert panels, therefore, recommend management of STS in tertiary reference centers and aim to standardize multidisciplinary care between them (Swallow et al. 2021; Gronchi et al. 2021; (TARPSWG) T-ARSWG 2018; ; ; ; ; Onkologie and (Deutsche Krebsgesellschaft DK, AWMF) 2022; Rothermundt et al. 2023). The first German guideline for STS in adults was published in September 2021 and updated in June 2022 (Onkologie and (Deutsche Krebsgesellschaft DK, AWMF) 2022, 2021). Based on these guidelines, the German Cancer Society (DKG, Deutsche Krebsgesellschaft) conducts regular audits to promote high-quality treatment standards (German Cancer Society (Deutsche Krebsgesellschaft) 2024). The certification of cancer centers by the DKG is a multistep process (Society GC 2024). Centers apply and conduct a self-assessment based on quality indicators generated by evidence-based guidelines of the DKG. Subsequently, independent sarcoma experts carry out on-site audits and file a detailed report after which they provide a recommendation for or against certification. The final decision is made by an independent panel of further sarcoma experts. Certified cancer centers must adhere to continuous quality improvements and create annual reports based on which the certificate is re-issued. Although this process ensures quality standards within each center, there are currently no analyses comparing decision-making between different centers in German-speaking countries. More specifically, it is currently unclear to what extent the decision-making process between centers is uniform. A recent study by Tirotta et al. investigating inter-center agreement in management of retroperitoneal STS across all 12 MTBs for retroperitoneal sarcoma in Great Britian revealed a clear inconsistency across centers (Tirotta et al. 2023b).

This study sought to investigate to what extent identical clinical cases are treated uniformly at different German-speaking sarcoma centers.

## Methods

In this cross-sectional case-based survey study, we distributed 5 anonymized patient cases of localized STS (2 retroperitoneal, 2 extremity, and 1 trunk wall) to 21 tertiary sarcoma centers from Germany (n = 20) and Austria (n = 1) including 20 of 21 sarcoma centers certified by the German Cancer Society at the time of the study (DKG). Patient characteristics are summarized in Table 1. Representative radiographic images can be found in the Supplementary Figs. 1–5. Specific details on each treatment modality, including surgical approaches, chemotherapy regimens, and radiotherapy dosing, are provided in Supplementary Table 1. For each case, data on patient characteristics, initial clinical presentation, imaging, and pathology data were sent to be discussed in the MTB of the participating centers. Patients were considered to have a good performance status, no limiting comorbidities, and a desire for maximum therapy. Participating centers were determined by literature and online search for tertiary sarcoma centers and initially recruited through email invitations on 31 October 2023. They were asked to present the cases, including all the aforementioned information in their MTB meetings. Cases were presented to the MTBs between November 2023 and April 2024. The participants were informed about the nature of the study, and the decision-making process was to be handled in the same way as a second opinion request. Participants were not informed about the actual treatments of each case and the decisions of other centers.

**Table 1:** Patient characteristics

Pat ID	Age and sex	Pathology diagnosis	Presentation status^a^	Location	Radiographic findings^b^
1	57 Male	DDLPS G2	Rapidly increasing swelling in the right groin within weeks	Right groin	MRI abdomen and pelvis with contrast: 19 cm craniocaudal measuring mass in the right iliopsoas muscle No bone or vascular infiltration CT chest and abdomen with contrast: no indication of metastases
2	67 Female	Myxoid liposarcoma G3	Four week history of increasing painless mass on the back	Right shoulder blade	MRI chest and shoulder with contrast: smoothly bordered oval mass (60 × 22 × 73 mm) on the right dorsal thoracic wall between the scapula and thoracic spine, directly overlying the posterior ribs intermuscularly between the serratus anterior, rhomboid and trapezius muscles No infiltration of neighboring structures CT chest and abdomen with contrast: No indication of metastases
3	68 Male	Locally recurrent DDLPS G2	Local recurrence on follow-up imaging. Primary diagnosis ten years earlier treated with resection only	Right retroperitoneum	CT chest and abdomen with contrast: locally recurrent 15 cm measuring mass with contact to the colon and ureter. No infiltration of the large vessels No infiltration of large vessels No indication of metastases
4	63 Male	WDLPS G1	Increasing digestive problems and gastric emptying disorders	Right retroperitoneum	CT chest and abdomen with contrast: 8 × 10 × 12 cm lipomatous tumor with intratumoral septa and broad-based contact to the right lower pole of the kidney, the duodenum and the iliac vessels on the right No evidence of intra-abdominal or thoracic metastases
5	69 Male	Pleomorphic rhabdomyosarcoma G3	Palpable mass in the right popliteal fossa	Right popliteal fossa	MRI right knee with contrast: Inhomogeneous formation in the right popliteal fossa (2.8 × 1.9 × 3.9 cm) with encirclement of the popliteal artery. No Baker's cyst PET-CT with contrast: No further tumor manifestations

*cm* centimeter, *CT* computed tomography, *DDLPS* dedifferentiated liposarcoma, *G* grade, *MRI* magnetic resonance imaging, *PET-CT* positron emission tomography–computed tomography, *WDLPS* well-differentiated liposarcoma

^a^All patients were considered to have a good performance status, no limiting comorbidities, and a desire for maximum therapy

^b^For representative images see supplementary files

Centers were then asked to fill out a written 45-item questionnaire with 9 items each case (Supplementary File 1). For each case, the survey contains 5 multiple choice and 4 open-ended question items. The questionnaire was written in German and designed to assess next steps in management, decision rationales, procedural details, the degree of consensus among participating specialists and the specialist disciplines present at the MTB meeting.

Specifically, centers were first asked in an open question to give their MTB recommendation. Secondly, centers were asked in a multiple-choice question if further diagnostics were necessary and if so, the specification of the necessary diagnostics. In the third and open question, the treatment recommendation was asked supposing there are no further diagnostics. The fourth and multiple-choice question covered all possible therapeutic modalities including surgery, chemotherapy, radiotherapy, regional hyperthermia, targeted therapies, clinical trial recruitment, best supportive care or other recommendations. Each therapeutic modality contained questions on specific details. Details on chemotherapy or targeted therapies included the agent, dosing, number of cycles and duration of each cycle. For surgery, details were asked on the extent of resection, the wound reconstruction as well as critical structures to be preserved or sacrificed during surgery. Details on radiotherapy covered the type of radiation, dose per fraction, fractions per day, total dose and technique. On regional hyperthermia questions focused on the concomitant treatment (chemotherapy or radiotherapy), number of sessions, sessions per week, duration of each session and target temperature. In the fifth question the sequence of the recommended therapy modalities from question four was asked. The sixth and open question asked for the rationale of the recommended procedure. In the seventh question the consensus among the participating specialists was assessed on a scale from 1 (lowest consensus) to 10 (highest consensus). The eighth question asked for the best alternative procedure and the ninth question for the specialist disciplines present at the MTB.

The responses to closed multiple-choice questions were analyzed and displayed descriptively in relation to the total number of responses. The responses to open-ended questions were analyzed through the following multi-step systematic approach: compilation of all responses into one dataset, categorization of responses, analysis of the frequency of response categories, and interpretation in relation to the topic of the question. Ambiguous answers or answers not addressing the topic of the questions were removed from the analysis. For each case, the treatment recommended by the highest number of centers was divided by the total number of recommendations to record the agreement percentage. Abstentions were reported and removed from the descriptive statistical analysis. GraphPad Prism v.9.3.1 (GraphPad Software, San Diego, CA, USA) was used for statistical analysis and figure design. The study was approved by the institutional review board of the Charité University Medicine Berlin (EA1/126/23).

The consensus level was determined for each case in ascending order on a scale of 1 to 10. In all 5 cases, recommendations by the 21 centers were categorized into three distinct levels of detail. In the first and most detailed category, treatment recommendations were analyzed based on the treatment modality and sequence of treatments (most detailed recommendations). Subsequently, the recommendations were grouped together based on recommendations with and without regional hyperthermia (grouped recommendations). Finally, recommendations were further grouped by aggregating pre- or postoperative treatment modalities into a unified perioperative category (most grouped recommendations).

## Results

In total, five cases were evaluated by 21 MTBs giving a total of 105 recommendations. There were no abstentions.

### Case 1: localized dedifferentiated liposarcoma (G2) in the right groin

**Fig. 1:**
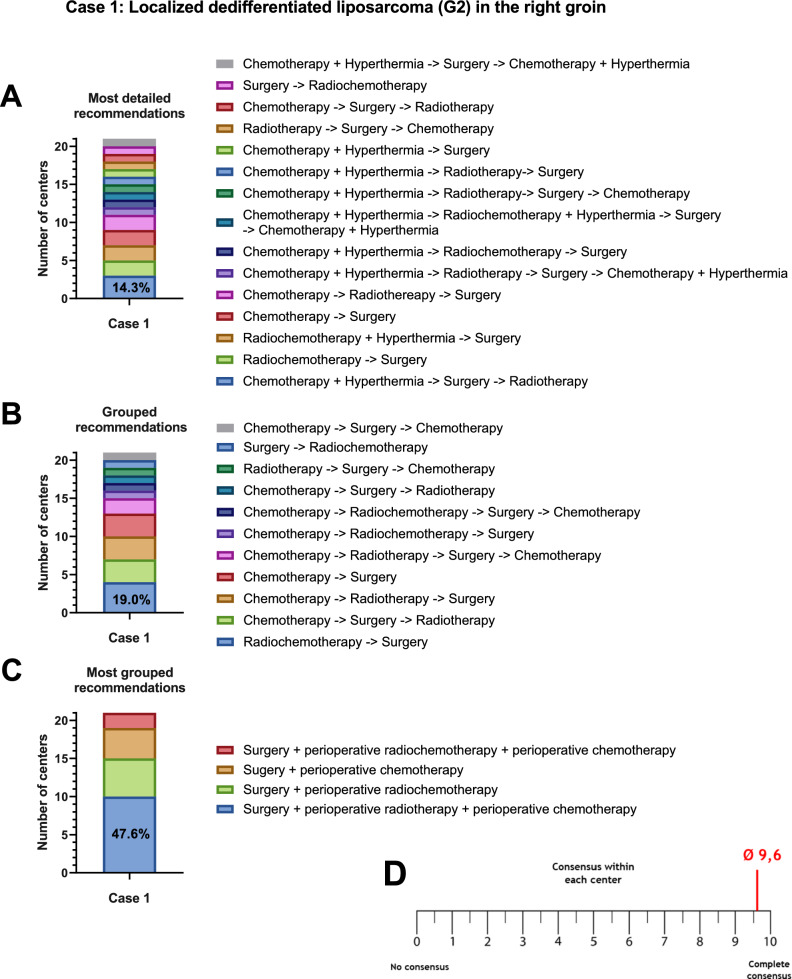
Analysis of recommendations for case 1: Localized dedifferentiated liposarcoma (G2) in the right groin. Detailed treatment recommendations are outlined, including specific therapy modalities and sequences (**A** most detailed recommendations). Recommendations are then categorized into groups with and without regional hyperthermia (**B** grouped recommendations). Finally, recommendations are further summarized by also combining preoperative and postoperative treatment modalities into “perioperative treatment modalities” (**C** most grouped recommendations). The degree of consensus is displayed in **D**

Out of 21 responding centers, 15 treatment recommendations were given (Fig. 1A). The most common recommendation with an agreement percentage of 14.3% from 3 centers was regional hyperthermia with chemotherapy, followed by surgical resection and postoperative radiotherapy. Grouping of recommendations with and without regional hyperthermia resulted in a total of 10 recommendations (Fig. 1B). The highest agreement was found with radiochemotherapy (with or without regional hyperthermia) followed by surgery favored by 4 centers (19.0%). Upon further grouping of cases of pre- or postoperative therapies to perioperative therapies, 4 different recommendations remained (Fig. 1C). The most common recommendation by 10 centers was surgery with perioperative radiotherapy and perioperative chemotherapy with or without regional hyperthermia (47.6% agreement). Five centers (23.8%) recommended surgery with perioperative radiochemotherapy, 4 (19%) surgery with perioperative chemotherapy, and 2 (9.5%) surgery with perioperative radiochemotherapy and chemotherapy. The mean level of consensus within each center was 9.6 (Fig. 1D).

### Case 2: localized myxoid liposarcoma (G3) in the right shoulder blade

**Fig. 2:**
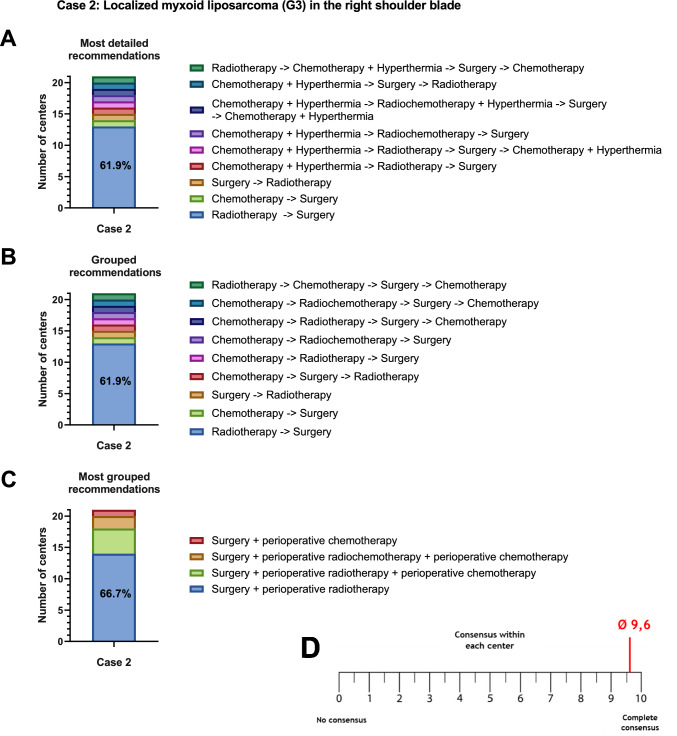
Analysis of recommendations for case 2: localized myxoid liposarcoma (G3) in the right shoulder blade. Detailed treatment recommendations are outlined, including specific therapy modalities and sequences (**A** most detailed recommendations). Recommendations are then categorized into groups with and without regional hyperthermia (**B** grouped recommendations). Finally, recommendations are further summarized by also combining preoperative and postoperative treatment modalities into “perioperative treatment modalities” (**C** most grouped recommendations). The degree of consensus is displayed in **D**

From 21 responding centers, 9 different recommendations were given (Fig. 2A). The most common recommendation by 13 centers was preoperative radiotherapy with subsequent surgical resection (61.9% percentage agreement). In the grouped analysis, 9 recommendations were given and the most common by 13 centers remained preoperative radiotherapy followed by surgery (61.9% agreement percentage, Fig. 2B). Further grouping yielded 4 different recommendations with surgery and perioperative radiotherapy by 14 centers as the most common (66.7%, Fig. 2C). Moreover, 4 centers (19%) recommended surgery with perioperative radiotherapy and perioperative chemotherapy, while 2 (9.5%) preferred surgery with perioperative radiochemotherapy and perioperative chemotherapy and 1 (4.8%) center decided for surgery and perioperative chemotherapy. The mean consensus level within the MTBs across all centers was 9.6 (Fig. 2D).

### Case 3: locally recurrent dedifferentiated liposarcoma (G2) in the right retroperitoneum

**Fig. 3:**
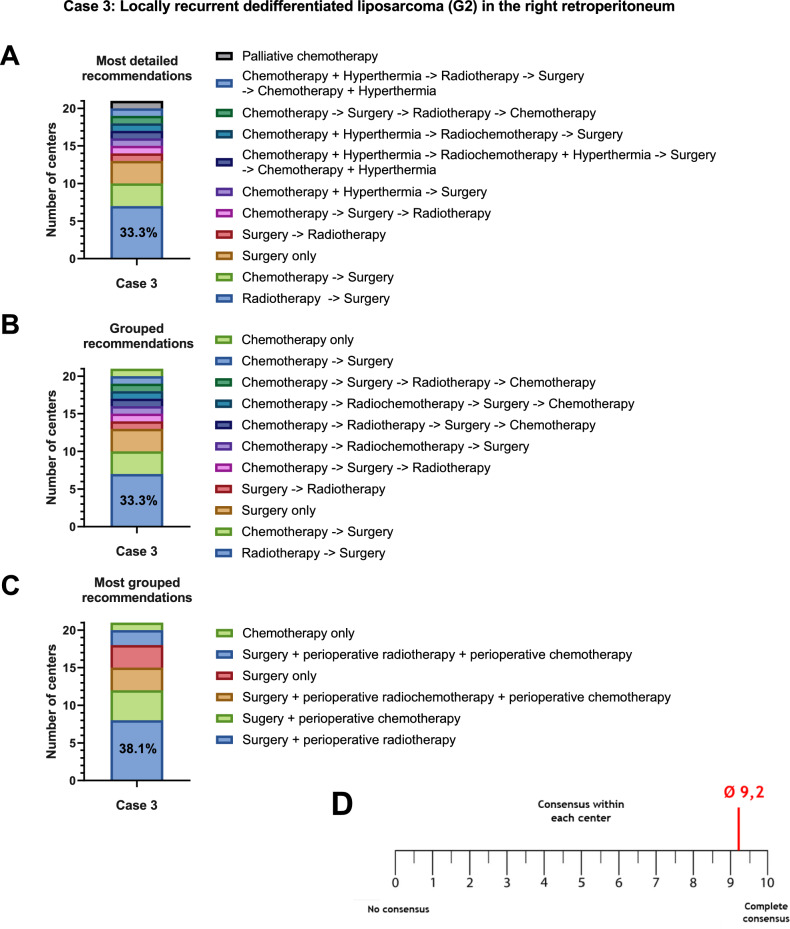
Analysis of recommendations for case 3: locally recurrent dedifferentiated liposarcoma (G2) in the right retroperitoneum. Detailed treatment recommendations are outlined, including specific therapy modalities and sequences (**A** most detailed recommendations). Recommendations are then categorized into groups with and without regional hyperthermia (**B** grouped recommendations). Finally, recommendations are further summarized by also combining preoperative and postoperative treatment modalities into “perioperative treatment modalities” (**C** most grouped recommendations). The degree of consensus is displayed in **D**

In total, 21 centers proposed 11 treatment recommendations (Fig. 3A). The most common recommendation by 7 centers was preoperative radiotherapy with subsequent surgical resection (33% percentage agreement, Fig. 3A). In the grouped analysis, preoperative radiotherapy and surgery remained the most common recommendation by 7 centers (33.3%). Upon further grouping, the most common recommendation was surgery with perioperative radiotherapy by 8 centers (38.1%). Further recommendations in the most grouped analysis were: 4 centers (19%) surgery and perioperative chemotherapy, 3 centers (14.3%) surgery and perioperative radiochemotherapy and perioperative chemotherapy, 3 centers (14.3%) surgery only, 2 centers (9.5%) surgery and perioperative radiotherapy and perioperative chemotherapy, and 1 center (4.8%) decided for palliative chemotherapy. The average consensus level within each MTB across all centers was 9.2 (Fig. 3D).

### Case 4: well-differentiated liposarcoma (G1) in the right retroperitoneum

**Fig. 4:**
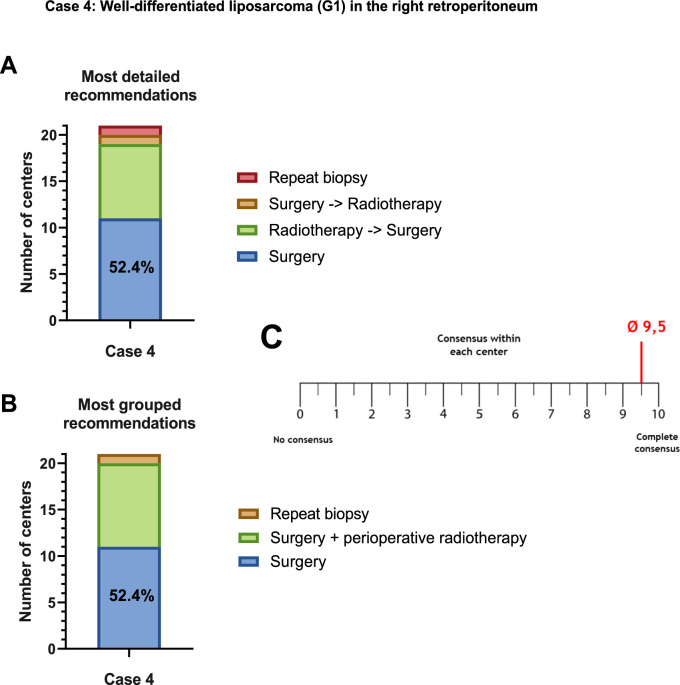
Analysis of recommendations for case 4: Well-differentiated liposarcoma (G1) in the right retroperitoneum. Detailed treatment recommendations are outlined, including specific therapy modalities and sequences (**A** most detailed recommendations). Since regional hyperthermia is not commonly used for management in well-differentiated G1 liposarcoma, no grouping for this treatment modality was necessary. Recommendations are summarized by combining preoperative and postoperative treatment modalities into “perioperative treatment modalities” (**B** most grouped recommendations). The degree of consensus is displayed in **C**

The 21 centers gave 4 different treatment recommendations (Fig. 4A). More than half of all centers (n = 11) decided for surgery only (52.4% agreement, Fig. 4A). One center has opted for a repeat biopsy. Since regional hyperthermia is not commonly used for management in well-differentiated G1 liposarcoma, no grouping for this treatment modality was necessary. When grouped for perioperative treatments, surgery consequently remained the most recommended modality preferred by 11 centers (52.4%, Fig. 4B). In contrast, 9 centers (42.8%) preferred surgery with perioperative radiotherapy and 1 center (4.8%) asked for a repeat biopsy. An average degree of consensus of 9.5 was reached (Fig. 4D).

### Case 5: localized pleomorphic rhabdomyosarcoma (G3) in the right popliteal fossa

**Fig. 5:**
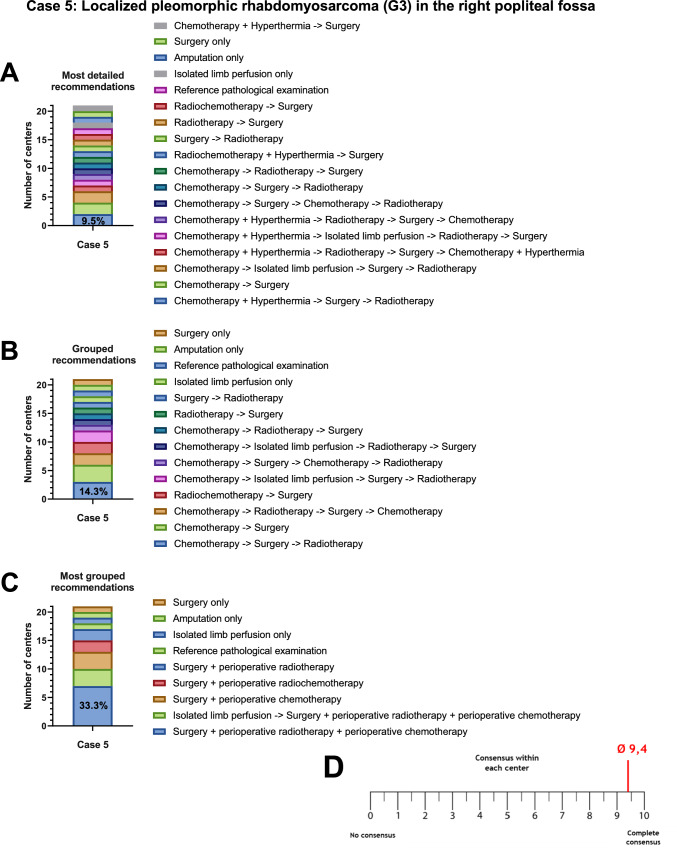
Analysis of recommendations for case 5: localized pleomorphic rhabdomyosarcoma (G3) in the right popliteal fossa. Detailed treatment recommendations are outlined, including specific therapy modalities and sequences (**A** most detailed recommendations). Recommendations are then categorized into groups with and without regional hyperthermia (**B** grouped recommendations). Finally, recommendations are further summarized by also combining preoperative and postoperative treatment modalities into "perioperative treatment modalities" (**C** most grouped recommendations). The degree of consensus is displayed in **D**

Case 5 evoked the highest number of different recommendations with 18 recommendations from 21 centers (Fig. 5A). The highest agreement in treatment recommendations was observed, with only 2 centers (9.5%) supporting each of the following approaches: preoperative chemotherapy combined with regional hyperthermia followed by surgery and postoperative radiotherapy; chemotherapy combined with surgery; and chemotherapy combined with isolated limb perfusion, surgery, and radiotherapy. While 1 center recommended isolated limb perfusion alone and 1 recommended reference pathological examination, the other 19 centers recommended curative therapies. One center favored amputation. After the first grouping, 14 therapy recommendations remained (Fig. 5B). Treatment consensus was limited, with 2 centers (14.3%) favoring preoperative chemotherapy (with or without regional hyperthermia) followed by surgery and postoperative radiotherapy, while an equal proportion supported chemotherapy followed directly by surgery. In the most grouped presentation, 9 different recommendations were shown (Fig. 5C). After grouping, the following recommendations remained: 3 centers (14.3%) for isolated limb perfusion followed by surgery with perioperative radiotherapy and perioperative chemotherapy (Fig. 5C), 3 centers (14.3%) for surgery with perioperative chemotherapy, 2 centers (9.5%) for surgery with perioperative radiochemotherapy, and 2 (9.5%) for surgery with perioperative radiotherapy. One center (4.8%) asked for reference pathological examination, while another (4.8%) suggested isolated limb perfusion only. Finally, 1 center (4.8%) recommended surgery only and 1 center (4.8%) decided for amputation. The largest consensus was among 7 centers (33.3%) that recommended surgery with perioperative radiotherapy and perioperative chemotherapy (with or without regional hyperthermia). The mean degree of consensus was 9.4 (Fig. 5D).

### Summative analysis

**Fig. 6:**
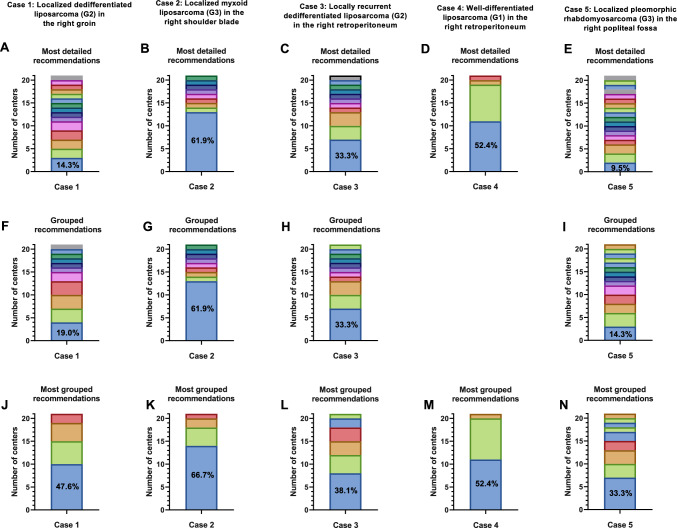
Overview of treatment recommendations for all 5 cases. Detailed treatment recommendations for case 1 to case 5 are outlined, including specific therapy modalities and sequences (**A**–**E** most detailed recommendations). Recommendations for all 5 cases are then categorized into groups with and without regional hyperthermia (**F**–**I** grouped recommendations). Case 4 is not represented, as regional hyperthermia is not applied in low-grade soft tissue sarcoma. Finally, recommendations for all 5 cases are further summarized by also combining preoperative and postoperative treatment modalities into "perioperative treatment modalities" (**J**–**N** most grouped recommendations)

The summative analysis of all 5 cases and recommendations is depicted in Fig. 6. In the most detailed recommendation analysis, the median agreement percentage across all 5 cases was 33.3%. In the grouped presentation, the median percentage agreement decreased slightly to 26.2%. Importantly, in the grouped presentation, case 4 was not represented, as regional hyperthermia is not applied in low-grade soft tissue sarcoma and thus no grouping can be applied. By aggregating data of pre- or postoperative therapies to “perioperative” therapies and grouping with and without regional hyperthermia in the most grouped analysis, the median percentage agreement rose to 47.6%. Moreover, the mean consensus level within each center across all 5 cases was 9.5. Targeted therapies were not recommended by any center for any of the five cases.

## Discussion

Herein, we present the first analysis on inter-center agreement across 21 German-speaking STS tertiary cancer centers based on five common cases of localized STS. Overall, the inter-center agreement on treatment allocation was low while the consensus within each center was very high.

A previous study investigating surgical aspects of retroperitoneal STS management across multiple centers specifically was recently published by Tirotta et al. (Tirotta et al. 2023a). The authors found striking differences in treatment recommendations for 21 cases presented to 12 centers in Great Britain. Most remarkably, in 11 out of 21 cases some centers recommended curative surgical approaches with or without perioperative treatments while others opted for palliative treatment recommendations. However, the study has important differences to our study which limit its comparability: firstly, it only included retroperitoneal STS. Secondly, the focus of the surgical colleagues was primarily on the question of resectability and extent of organ resections and then on the recommended treatment modalities. Within the cases that were deemed resectable by most or all centers, the recommendation was quite uniformly upfront surgery in most cases. The treatment allocations started to diverge into different groups (curative vs. palliative) within the groups where the resectability was not uniformly clear. The authors therefore called for establishing specific resectability criteria in retroperitoneal STS and concluded that more standardization is necessary and that further exchange of MTBs across different centers may be necessary to achieve this (Tirotta et al. 2023a). In our study, except for two exceptions, no center deemed any case unresectable and thus almost all treatment allocations were with curative intention. Moreover, we included 2 retroperitoneal, 2 extremity, and 1 trunk wall STS. The focus of our study was rather on assessing the inter-center agreement on treatment modalities and sequences across all participating specialties for different clinical scenarios at MTBs. A similar study for non-small cell lung cancer was recently conducted on 10 patient cases at 11 MTBs in the Netherlands and revealed a broad variety of both clinical staging and subsequent treatment recommendation (Hoeijmakers et al. 2020). Still, what unites the previous two and the present study is the call for exchange between the centers MTBs to drive standardization beyond the level of guidelines only.

There are many underlying reasons possibly explaining the remarkable non-uniformity across sarcoma centers presented herein. The first and foremost is scarcity of clinical data. Particularly on questions of treatment sequencing there is often no head-to-head data comparing one to another. It can also be argued that the exact distinction between the treatment sequences may not make a strong clinical difference and thus, for example, the recommendation for pre- or postoperative chemotherapy can be summarized as “perioperative chemotherapy”. This grouping of treatments would reduce the display of variability between the centers. Nevertheless, as shown in Fig. 6J–N, a considerable dispersion of treatment recommendations with a median percentage agreement of 47.6% still remains.

Another reason may be center-specific conventions and routines, which have established themselves over time and favor a certain treatment combination or sequence without unequivocal underlying evidence for or against it. Finding supportive scientific evidence for this hypothesis is challenging. Still, the authors of this project consider center-specific routines preferring certain treatments and sequences as a very common real-world situation at the 21 different centers where they practice. This is also partly reflected in the very high degrees of consensus within each center with an average of 9.5 across all centers that uniformly agrees on their own treatment recommendations. This observation provides another rationale for the idea of nation- or region-wide MTBs to challenge the structures from one center with views from other centers. In this way, own structures and routines can be questioned, new ideas introduced, and ultimately the standardization of therapy enforced beyond guidelines only. In addition, the ring trial described herein can be further elaborated and used in a systematic form as a regular quality assurance measure between the centers.

This and the previous studies are among the first to analyze the agreements in treatment allocation between tertiary sarcoma centers (Tirotta et al. 2023a; Hoeijmakers et al. 2020). Although inter-center agreement is significantly lower than would be expected, it may be that this degree of variability ultimately reflects clinical reality. Importantly, guidelines are not obligatory directives and it is up to the expertise of the MTB and its specialists to decide which therapy is most appropriate for the patient. Nevertheless, the question then arises as to the extent to which center-specific variations are acceptable and when the deviations are too great. Especially, in situations such as in the British study by Tirotta et al., in which a large proportion recommended curative and another palliative therapy, as the consequences for the individual patient are obviously very great (Tirotta et al. 2023a).

### Case-by-case discussion

It is important to review the cases individually. Case 1 demonstrates an estimated 5-year survival of 69% according to the Sarculator (Callegaro et al. 2016; Pasquali et al. 2019). This surpasses the threshold suggesting perioperative chemotherapy's advantage in high-risk metastatic settings, as indicated in the posthoc analysis of the recent EORTC trial on adjuvant therapy (Woll et al. 2012). Regarding radiotherapy sequencing, the inherent higher risk of wound healing complications supports consideration of preoperative radiotherapy; however, the potential for subsequent complications may argue for postoperative radiotherapy (O'Sullivan et al. 2002, 2004; Haas et al. 2012). Herein, the most frequent recommendation by 3 centers only was preoperative chemotherapy followed by surgery and postoperative radiotherapy (Fig. 1A).

In case 2, the feasibility of the DOREMY protocol with reduced radiation dose favors preoperative radiotherapy (Lansu et al. 2021; Roohani et al. 2024). Similar findings were confirmed in another prospective trial where preoperative short-course hypofractionated radiotherapy followed by wide resection let to excellent local control rates (Koseła-Paterczyk et al. 2020). Comprehensive familiarity with current literature on STS is essential in decision-making, although it remains an open question whether an universal and comprehensive knowledge on STS care can be expected across all certified centers. The use of perioperative chemotherapy may be influenced by tumor chemosensitivity and the potential for less toxic therapy with trabectedin compared to doxorubicin and ifosfamide (Pasquali et al. 2019; Gronchi et al. 2017). Still, most likely due to the excellent outcomes reported in the DOREMY trial, this case exhibited the highest level of consensus among all cases, with 13 out of 21 centers opting for preoperative radiotherapy followed by surgery (Fig. 2A).

Cases 3 and 4 reflect the STRASS and STREXIT debate concerning the use of preoperative radiotherapy for retroperitoneal STS (Roohani et al. 2024; Bonvalot et al. 2020; Callegaro et al. 2023; Haas et al. 2019). While the randomized study yielded negative results for an unselected group of retroperitoneal STS, positive subgroup analyses for liposarcomas (well-differentiated liposarcomas and grade 1 and 2 dedifferentiated liposarcomas) underscore its potential efficacy. However, locally recurrent disease has not been investigated in prospective studies, yet. Until the prospective STRASS data is updated, this topic often sparks vigorous debate among proponents of radiotherapy with surgery versus surgery only as it is depicted by the great variability among centers with 7 out of 21 being the highest numbers for preoperative radiotherapy and surgery in case 3 (Fig. 3A), and 11 out of 21 for surgery only in case 4 (Fig. 4A).

In case 5, the histology would generally be viewed as a high-grade adult-type STS, where preoperative radiotherapy followed by wide resection is recommended (Gronchi et al. 2021; Salerno et al. 2021). Alternative treatments such as isolated limb perfusion or combined chemotherapy and hyperthermia warrant consideration, although availability and proximity to specialized centers remain significant factors (Jakob and Hohenberger 2016; Hayes et al. 2024; Issels et al. 2010). The decision shows considerable variability and lack of uniformity, with recommendations ranging widely (Fig. 5A). The highest level of consensus observed is 3 out of 21 centers (14.3%) advocating for preoperative chemotherapy and hyperthermia, followed by surgery and then postoperative radiotherapy (Fig. 5A).

These arguments are likely topics of discussion in every center. The contentious results of the ring trial were presented at the German Sarcoma Conference, fostering a professional exchange that enhances the individual strength of centers and the network of German-speaking sarcoma centers. Laboratories routinely conduct and repeat ring trials as part of quality control measures. We have demonstrated the feasibility of conducting a ring trial for tumor boards with high voluntary participation rates. Continuing this project has the potential to enhance sarcoma patient care.

### Strength and limitations

The strengths of our study are its novelty, and validity. Apart from the study by Tirotta et al. which rather focused on surgical aspects and resectability criteria for retroperitoneal STS only, our study is the first one to dedicatedly compare multidisciplinary and inter-center treatment recommendations for different, yet common clinical scenarios of curable, localized STS. All centers were provided with the original radiological imaging data to assess the clinical scenario more realistically and comprehensively. This allowed for a more thorough evaluation than is typically possible during conferences, where only a limited number of images are presented in a brief session. In addition, with 21 participating centers in German-speaking countries, including 95.2% (20 out of 21) of DKG-certified STS centers, the study has strong external validity and representation of clinical practice on STS management at major cancer centers in German-speaking countries. By focusing on German-speaking cancer centers, we minimized the potential influence of cultural differences and variations in training as underlying factors for differing treatment recommendations. This approach constitutes another strength of our study.

However, the study must also be considered in light of the following limitations: Firstly, the number of five presented cases is low. Higher case numbers and more diverse cases may reflect the clinical reality more precisely. The small sample size impedes robust statistical analysis of the degree of agreement among centers. Secondly, the disseminated survey is not a validated tool. Instead, the questionnaire rather aimed to cover very practical and concrete questions on the management of the presented cases. Thirdly, participating specialists in the MTBs were informed about the study in beforehand. They were aware that their answers would be analyzed together with surveys from other centers and their decisions would not have the same clinical consequences as real cases. Lastly, the information available to the MTB was limited to what was sent to them for each case. In clinical reality, ambiguous findings would entail additional diagnostics in order to make a more appropriate decision. At the same time, it is important to mention that the request for further diagnostics was only made in very few of the cases presented.

## Conclusion

This is the first case-based analysis on inter-center agreement for STS management in German-speaking countries since publication of the first German guideline. Our study includes a remarkable number of DKG-certified sarcoma centers, covering nearly all tertiary centers in German-speaking countries, strongly affirming its external validity. The inter-center agreement in treatment allocation and sequence was low. At the same time, the MTBs within each center were very confident in their decisions as displayed by the high degrees of consensus. These findings may potentially indicate clinically very relevant differences in treatment standards among sarcoma centers. To harmonize treatment standards, ongoing research on new treatment modalities for STS patients is indispensable. Furthermore, additional case-based exchange between centers should be carried out to reduce the strong discrepancies in clinical reality presented herein and to further standardize the management of STS patients.

## Supplementary Information

Below is the link to the electronic supplementary material.

Supplementary file1 (TIF 471 KB)

Supplementary file2 (TIF 325 KB)

Supplementary file3 (TIF 540 KB)

Supplementary file4 (TIF 482 KB)

Supplementary file5 (TIF 298 KB)

Supplementary file6 (TIF 435 KB)

Supplementary file7 (TIF 277 KB)

Supplementary file8 (TIF 512 KB)

Supplementary file9 (TIF 590 KB)

Supplementary file10 (TIF 512 KB)

Supplementary file11 (TIF 518 KB)

Supplementary file12 (DOCX 30 KB)

Supplementary file13 (DOCX 80 KB)

## Data Availability

Data available on request from the corresponding author.

## Abbreviations

Cm: Centimeter
CT: Computed tomography
DDLPS: Dedifferentiated liposarcoma
DKG: German Cancer Society (Deutsche Krebsgesellschaft)
G: Grade
MRI: Magnetic resonance imaging
MTB: Multidisciplinary tumor board
PET-CT: Positron emission tomography–computed tomography
STS: Soft tissue sarcoma
WDLPS: Well-differentiated liposarcoma
