# Supplementary figures and images for: The sarcoma ring trial: a case-based analysis of inter-center agreement across 21 German-speaking sarcoma centers
Source: J Cancer Res Clin Oncol. 2025 Jan 4;151(1):30. doi: 10.1007/s00432-024-06063-z (PMC11700044; doi:10.1007/s00432-024-06063-z)

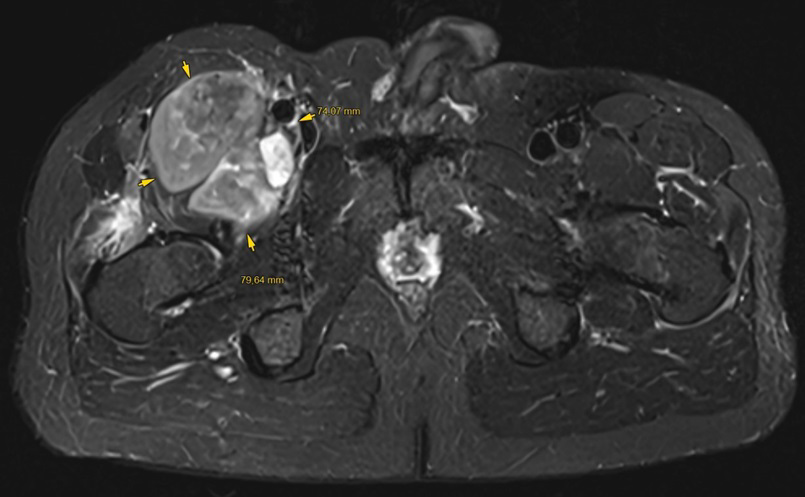

Supplement: Supplementary file 1 — Supplementary file1 (TIF 471 KB) [file 432_2024_6063_MOESM1_ESM.tif]

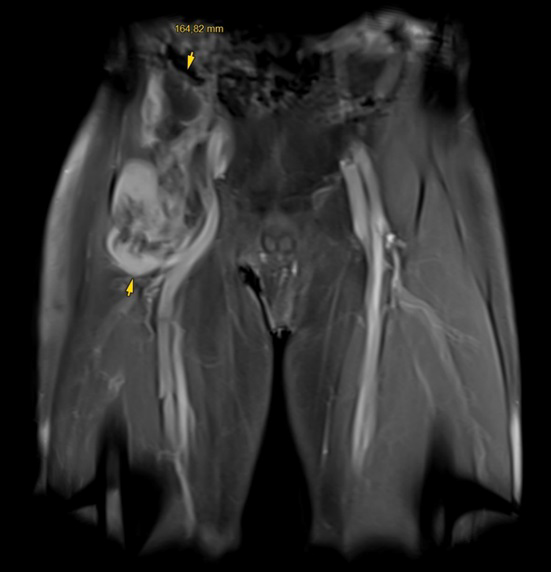

Supplement: Supplementary file 2 — Supplementary file2 (TIF 325 KB) [file 432_2024_6063_MOESM2_ESM.tif]

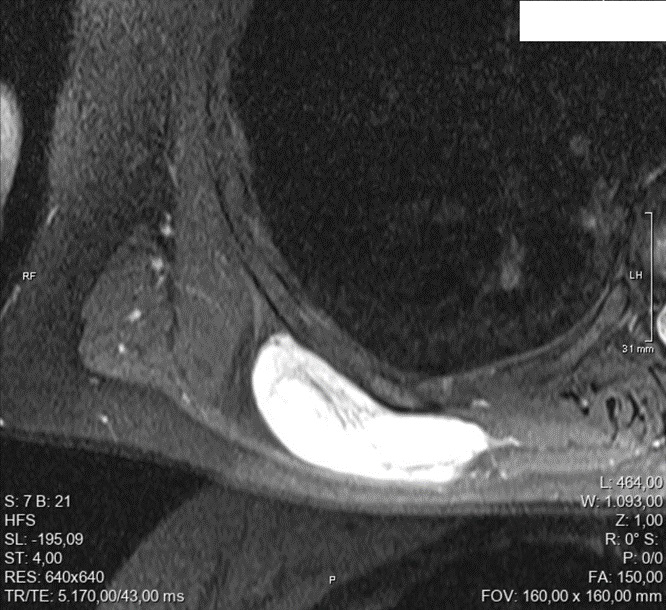

Supplement: Supplementary file 3 — Supplementary file3 (TIF 540 KB) [file 432_2024_6063_MOESM3_ESM.tif]

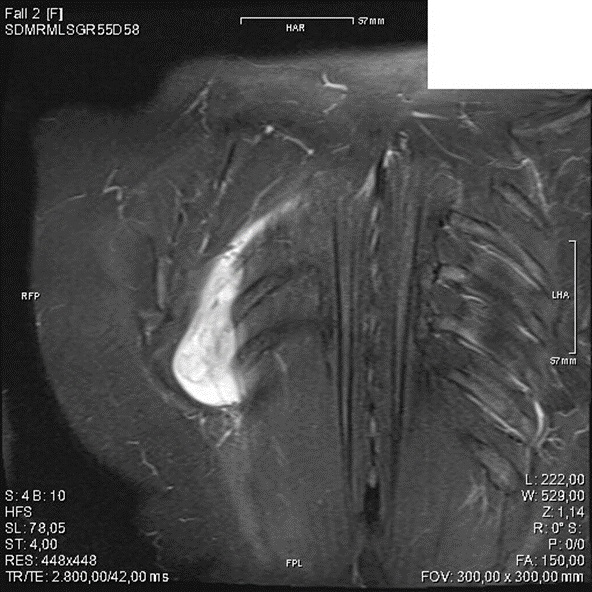

Supplement: Supplementary file 4 — Supplementary file4 (TIF 482 KB) [file 432_2024_6063_MOESM4_ESM.tif]

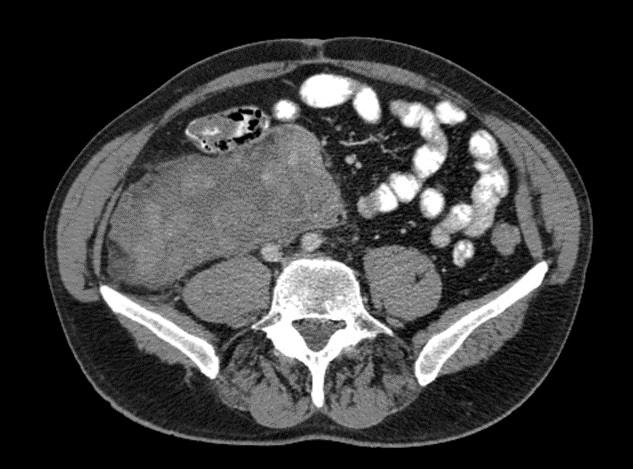

Supplement: Supplementary file 5 — Supplementary file5 (TIF 298 KB) [file 432_2024_6063_MOESM5_ESM.tif]

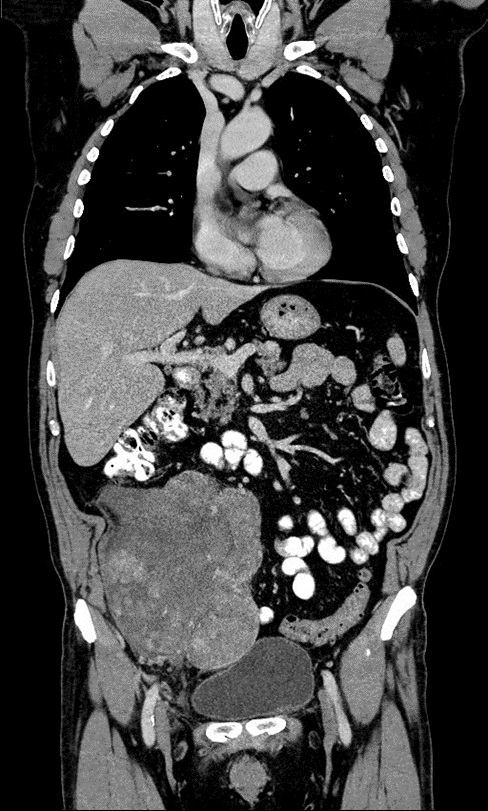

Supplement: Supplementary file 6 — Supplementary file6 (TIF 435 KB) [file 432_2024_6063_MOESM6_ESM.tif]

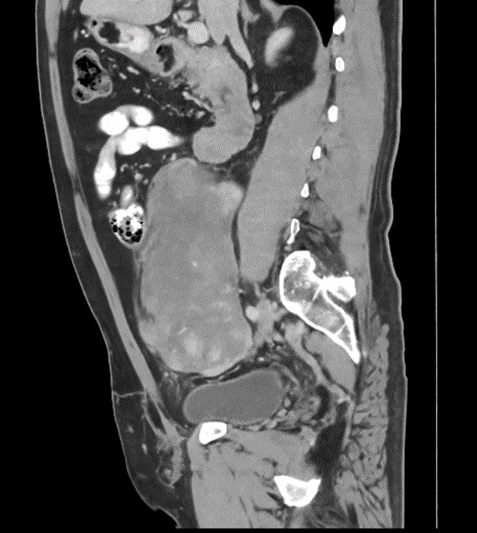

Supplement: Supplementary file 7 — Supplementary file7 (TIF 277 KB) [file 432_2024_6063_MOESM7_ESM.tif]

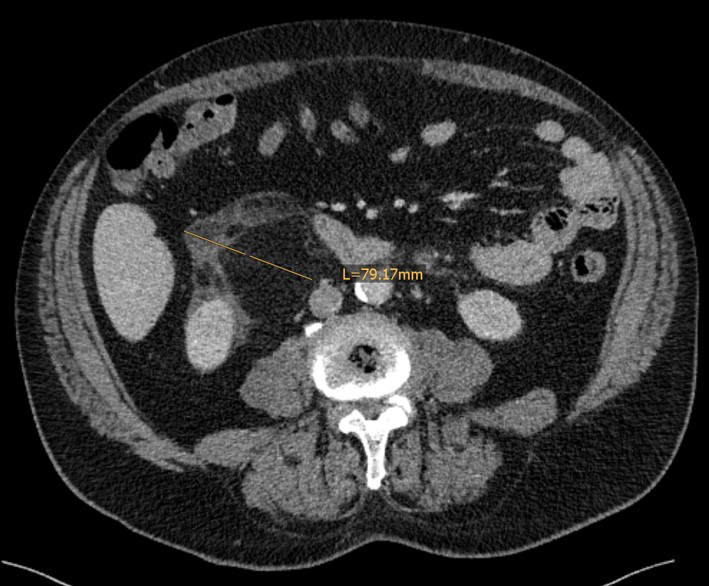

Supplement: Supplementary file 8 — Supplementary file8 (TIF 512 KB) [file 432_2024_6063_MOESM8_ESM.tif]

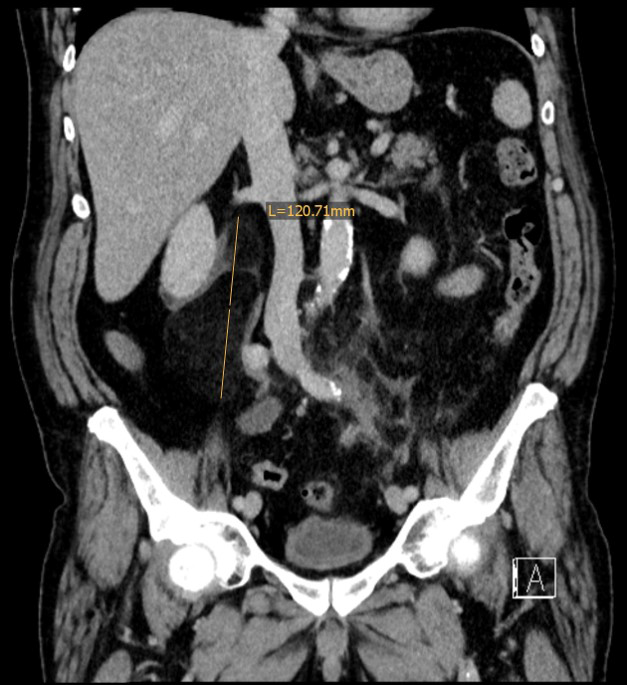

Supplement: Supplementary file 9 — Supplementary file9 (TIF 590 KB) [file 432_2024_6063_MOESM9_ESM.tif]

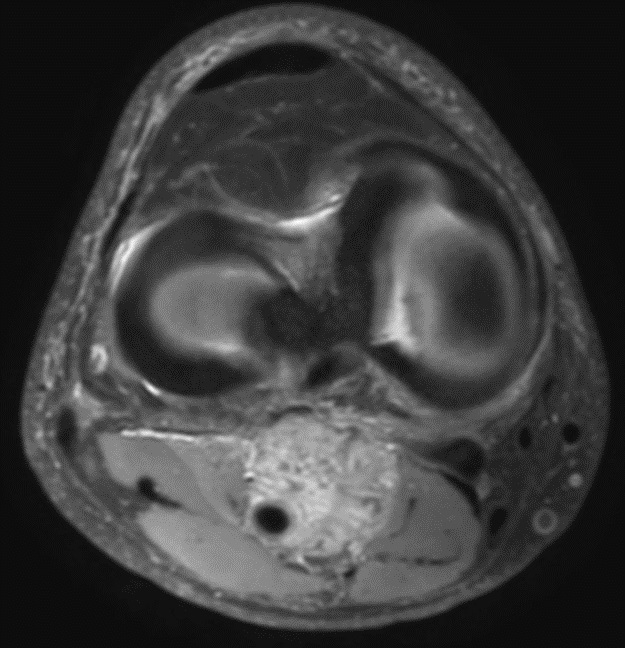

Supplement: Supplementary file 10 — Supplementary file10 (TIF 512 KB) [file 432_2024_6063_MOESM10_ESM.tif]

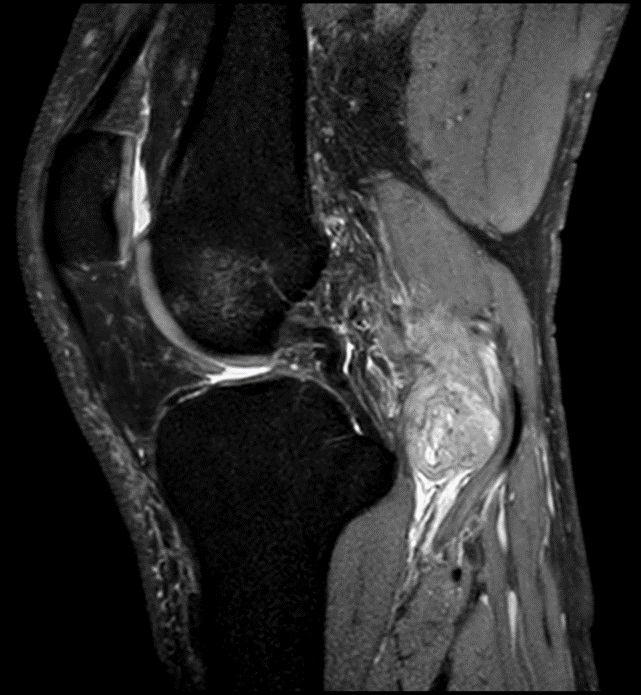

Supplement: Supplementary file 11 — Supplementary file11 (TIF 518 KB) [file 432_2024_6063_MOESM11_ESM.tif]
