# Supplementary material for: The sarcoma ring trial: a case-based analysis of inter-center agreement across 21 German-speaking sarcoma centers
Source: J Cancer Res Clin Oncol. 2025 Jan 4;151(1):30. doi: 10.1007/s00432-024-06063-z (PMC11700044; doi:10.1007/s00432-024-06063-z)
Supplement: Supplementary file 12 — Supplementary file12 (DOCX 30 KB) [file 432_2024_6063_MOESM12_ESM.docx]

Sarcoma Ring Trial

Please provide as a multidisciplinary tumor board a recommendation for further management or the next proposed therapy as free text and as a multiple-choice option with multiple selections assuming it is a localized disease and patients with maximal therapy desire and without limiting comorbidities.

Please provide a brief clinical rationale for your decision, e.g., with reference to scientific publications or clinic-specific prior experiences.

Name of completing center:

| 1. Please provide your tumor board recommendation as free text |  | | |
| --- | --- | --- | --- |
| 1. Further diagnostics required? | ☐ No | | |
|  | ☐ Yes, namely (multiple choices possible): | | |
|  | ☐ Repeat imaging | | ☐ PET-CT |
|  | ☐ Re-biopsy | | ☐ Further pathological diagnostics |
|  | ☐ Reference pathological assessment | | ☐ Molecular pathology / panel diagnostics |
|  | Other (free text): | | |
| 1. What are your therapy recommendations at this point without further diagnostics (free text)? |  | | |
| 1. What are your therapy recommendations at this point without further diagnostics? (multiple choices possible)? | ☐ Resection. If yes, please specify:  ☐ Wide resection with primary wound closure  ☐ Wide resection plus plastic reconstruction  ☐ Wide resection with marginal resection at critical structures (e.g., sciatic nerve)  If yes, which ones:  ☐ Wide resection with resection and if necessary replacement of critical structures (e.g., femoral artery)  If yes, which ones: | | |
|  | ☐ Chemotherapy (=CTX)   - Agent: - Dosage: - Number of cycles: | | |
|  | ☐ Radiotherapy (=RT):   - Type of radiation (Photons, Protons, etc.): - Dosage per fraction: - Fractions per day: - Total dosage: - Technique: | | |
|  | ☐ Deep Regional Hyperthermia (=HT)  ☐ Concurrent with chemotherapy  ☐ Concurrent with radiation therapy   - Total number of sessions: - Number of sessions per week: - Duration per session: - Target temperature: | | |
|  | ☐ Targeted Therapy (=TT)  • Agent:  • Dosage:  • Number of administrations: | | |
|  | ☐ Best supportive care | | |
|  | ☐ Enrollment in the following trial: | | |
|  | ☐ Other therapy recommendation (free text): | | |
| 1. Multimodal Therapy:   If you have recommended multimodal therapy, please provide the planned sequence | E.g. CTX +/- HT – RT - OP – CTX | | |
| 1. What is your rationale for this procedure (free text)? |  | | |
| 1. How much consensus was reached among all participants in this decision? (on a scale from 1 (lowest) to 10 (highest)) | 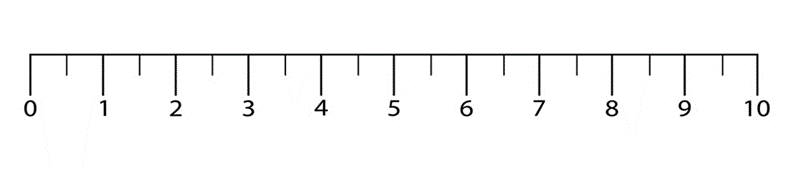 | | |
| 1. Best alternative procedure (Free text) ? |  | | |
| 1. Disciplines present at the tumor board meeting (Multiple choices possible) | ☐ General and visceral surgery | ☐ Orthopedic and trauma surgery | |
|  | ☐ Radiation oncology | ☐ Medical oncology | |
|  | ☐ Pathology | ☐ Neuropathology | |
|  | ☐ Radiology (including neuroradiology) | ☐ Nuclear medicine | |
|  | ☐ Vascular surgery | ☐ Plastic surgery | |
|  | ☐ Neurosurgery | ☐ Gynecology | |
|  | ☐ Otorhinolaryngology | ☐ Oral and maxillofacial surgery | |
|  | ☐ Urology | ☐ Anesthesiology | |
|  | ☐ Psycho-oncology | ☐ Social services | |
