# Supplementary material for: The sarcoma ring trial: a case-based analysis of inter-center agreement across 21 German-speaking sarcoma centers
Source: J Cancer Res Clin Oncol. 2025 Jan 4;151(1):30. doi: 10.1007/s00432-024-06063-z (PMC11700044; doi:10.1007/s00432-024-06063-z)
Supplement: Supplementary file 13 — Supplementary file13 (DOCX 80 KB) [file 432_2024_6063_MOESM13_ESM.docx]

|  | **Case 1: Localized dedifferentiated liposarcoma (G2) in the right groin** | | | | | | | |
| --- | --- | --- | --- | --- | --- | --- | --- | --- |
|  | **Surgery** | | | | **Chemotherapy (CTX)** | | | |
| Center | Wide resection with primary wound closure | Wide resection with plastic reconstruction | Wide resection with marginal resection at critical structures | Wide resection with replacement of critical structure | Agent | Dosage | No. of cycles | Duration of each cycle |
| 1 | Details N/A* | Details N/A* | Details N/A* | Details N/A* | AI | 75mg/m²  10g/m² | 6 | 3 weeks |
| 2 | - | - | - | Femoral artery | AI | 75mg/m² d1  1.5g/m²  d1-5 | 3 | 3 weeks |
| 3 | - | - | - | Femoral artery and vein | AI | 60mg/m² d1  3g/m² d1-3 | AI: 4  Ifo (+ RT): 2 | N/A |
| 4 | - | - | Vessels and epineurotomy of femoral nerve | - | AI | 60mg/m² 3x3g | 3 -> Re-Staging  -> 3 | 3 weeks |
| 5 | Details N/A* | Details N/A* | Details N/A* | Details N/A* | AI | 60mg/m² 9g/m² | 4 | 3 weeks |
| 6 | - | - | - | Femoral artery, proximal femoral replacement for tumor contact | AI | 60mg/m² d1 3g/m² d1-3 | 3 ->  Re-Staging -> 3 | 3 weeks |
| 7 | Details N/A* | Details N/A* | Details N/A* | Details N/A* | AI | 75mg/m² d1  1.5g d1-4 | 2 ->  Re-Staging -> 2 | 3 weeks |
| 8 | Details N/A* | Details N/A* | Details N/A* | Details N/A* | AI or Doxo/DTIC | N/A | 4-6 | 3 weeks |
| 9 | - | - | Femoral artery + femoral nerve | - | AI | 75mg/m²  1.5g/m² | 6 | 3 weeks |
| 10 | - | - | Femoral artery + femoral nerve | - | AI | 75mg/m²  7,5g/m² | 2 ->  Re-Staging -> 4 | 3 weeks |
| 11 | - | - | - | Femoral vessels, proximal femur (depends on re-staging) | AI | 75mg/m²  9g/m² | 5 | 3 weeks |
| 12 | - | - | - | Femoral artery and vein | AI | 60mg/m² d1  1.8g/m² d1-5 | 4 | 4 weeks |
| 13 | Yes | - | - | - | AI | 60mg/m² d1 2.5g/m² d1-4 | 5 | 3 weeks |
| 14 | - | - | - | Superficial femoral artery and vein | AI | 30mg/m² d1,2  2g/m² d1-5 | 6 | N/A |
| 15 | - | - | Femoral vessels and nerves | - | Epi/Ifo | 60mg/m² d1,2 3000 mg/m² d1-3 | - | - |
| 16 | Details N/A* | Details N/A* | Details N/A* | Details N/A* | AI | 75mg/m² d1 2500mg/m² d1-4 | 6 | N/A |
| 17 | Details N/A* | Details N/A* | Details N/A* | Details N/A* | AI | 60mg/m²  6-9g/m² | 8 | N/A |
| 18 | Details N/A* | Details N/A* | Details N/A* | Details N/A* | Doxo | N/A | 2 | N/A |
| 19 | - | - | - | Femoral artery and vein | AI | 75mg/m²  9g/m² | 2 -> Re-Staging -> 6 | 3 weeks |
| 20 | - | - | - | Depending on Re-staging | AI | 50-75mg/m² d1  15-2.5g d1-4 | 6 | 4 weeks |
| 21 | - | - | - | Femoral artery and vein. Depending on intraoperative findings: superficial femoral artery, femoral nerve branches | AI | 75mg/m² d1  2.5g/m² d1-4 | 2 -> Re-Staging | 3 weeks |
| ^*^Center recommends surgery, details on surgery are not available.  **Abbreviations/symbols:** - = Indicates that the center did not select this option. AI = Doxorubicin and Ifosfamide; CTX = Chemotherapy; d= day; Doxo= Doxorubicin; DTIC= Dacarbazine; Epi = Epirubicin; Ifo= Ifosfamide; N/A = Information not available; RT = Radiotherapy | | | | | | | | |

|  | **Case 1: Localized dedifferentiated liposarcoma (G2) in the right groin** | | | | | | | |
| --- | --- | --- | --- | --- | --- | --- | --- | --- |
|  | Radiotherapy (RT) | | | | Deep regional hyperthermia | | | |
| Center | Type of radiation | Dosage per fraction, fractions per day | Total dosage | Technique | Concurrent with  CTX or RT | No. of sessions per time -> total no. of sessions | Duration per session [min] | Target temperature [°C] |
| 1 | - | - | - | - | CTX | 2/cycle -> 12 | 90 | 42 |
| 2 | Photon | 2 Gy, once daily | 50 Gy | VMAT | - | - | - | - |
| 3 | Photon | 2 Gy, once daily | 66 Gy | IMRT | - | - | - | - |
| 4 | Photon | 2 Gy, once daily | 50 Gy | IMRT | - | - | - | - |
| 5 | Photon | 2 Gy, once daily | 50 Gy | IMRT | CTX and RT | 1/week (with CTX)  3/week (with RT)  -> 14 | 60 | 42-44 |
| 6 | Photon | 2 Gy, once daily | 60-66 Gy | IMRT/VMAT/IGRT | CTX | 2/week -> 12 | 90 | 43°C |
| 7 | - | - | - | - | - | - | - | - |
| 8 | - | - | - | - | CTX | N/A | N/A | N/A |
| 9 | Photon | 2 Gy, once daily | 50 Gy | IMRT | - | - | - | - |
| 10 | Photon | 2 Gy, once daily | 50 Gy | IMRT | - | - | - | - |
| 11 | Photon | 1.8 Gy, once daily | 50.4 Gy | IMRT/IGRT | CTX and RT | N/A | N/A | N/A |
| 12 | - | - | - | - | CTX | N/A | N/A | N/A |
| 13 | Photon | 1.8-2 Gy, once daily | 50-50.4 Gy | IMRT/VMAT | - | - | - | - |
| 14 | Photon | N/A | N/A | N/A | - | - | - | - |
| 15 | Photon | 2 Gy, once daily | 50 Gy | IMRT | - | - | - | - |
| 16 | Photon or proton | 2 Gy, once daily | 64 Gy | IMRT/VMAT | CTX | N/A | N/A | N/A |
| 17 | Photon | 2 Gy, once daily | 50 Gy | VMAT | CTX | 2/cycle -> 16 | 90 | 41 |
| 18 | - | - | - | - | - | - | - | - |
| 19 | Photon | 2 Gy, once daily | 50 Gy | IMRT/IGRT | CTX | 2/week -> 12 | N/A | N/A |
| 20 | Photon | 2 Gy, once daily | 54-60 Gy | VMAT/IGRT/SGRT | CTX and RT | 1-2/week -> 16 | 60 | 40-44 |
| 21 | Photon | 2 Gy, once daily | 50 Gy | IMRT/VMAT/IGRT | Details N/A* | Details N/A* | Details N/A* | Details N/A* |
| ^*^Center recommends regional hyperthermia, details on hyperthermia are not available.  **Abbreviations/symbols:** - = Indicates that the center did not select this option. CTX = Chemotherapy; d= day, IGRT = Image-guided radiation therapy; IMRT = Intensity modulated radiotherapy; N/A = Information not available; RT = Radiotherapy; SGRT = Surface Guided Radiation Therapy; VMAT = Volumetric Modulated Arc Therapy | | | | | | | | |

|  | **Case 2: Localized myxoid liposarcoma (G3) in the right shoulder blade** | | | | | | | |
| --- | --- | --- | --- | --- | --- | --- | --- | --- |
|  | **Surgery** | | | | **Chemotherapy (CTX)** | | | |
| Center | Wide resection with primary wound closure | Wide resection with plastic reconstruction | Wide resection with marginal resection at critical structures | Wide resection with replacement of critical structure | Agent | Dosage | No. of cycles | Duration of each cycle |
| 1 | Details N/A* | Details N/A* | Details N/A* | Details N/A* | Doxo/DTIC | 75mg/m²  1.2g/m² | 6 | 3 weeks |
| 2 | - | - | - | Partial rib resection and thoracic closure using bovine pericardium | - | - | - | - |
| 3 | - | Yes | - | - | - | - | - | - |
| 4 | Yes | - | - | - | - | - | - | - |
| 5 | Details N/A* | Details N/A* | Details N/A* | Details N/A* | AI | 60mg/m² 9g/m² | 4 | 3 weeks |
| 6 | - | Chest wall reconstruction | - | - | AI | 60mg/m² d1 3g/m² d1-3 | 3 ->  Re-Staging -> 3 | 3 weeks |
| 7 | Details N/A* | Details N/A* | Details N/A* | Details N/A* | - | - | - | - |
| 8 | Yes | - | - | - | - | - | - | - |
| 9 | Yes | - | - | - | - | - | - | - |
| 10 | Yes | - | - | - | - | - | - | - |
| 11 | Yes | - | - | - | - | - | - | - |
| 12 | - | - | - | Depending on intraoperative finding: thoracic wall resection | - | - | - | - |
| 13 | Yes | - | - | - | - | - | - | - |
| 14 | - | - | Chest wall, scapula | - | - | - | - | - |
| 15 | - | - | Possibly ribs, depending on tumor response to RT | - | - | - | - | - |
| 16 | Yes | - | - | - | Doxo/DTIC or trabectedin | 75mg/m² d1 400mg/m² d1-3 d1-4  1.5mg/m² | 6 | N/A |
| 17 | Yes, depending on tumor response to neoadjuvant treatments | Yes, depending on tumor response to neoadjuvant treatments | Yes, depending on tumor response to neoadjuvant treatments | Yes, depending on tumor response to neoadjuvant treatments | AI | 60mg/m²  6g/m² | 8 | N/A |
| 18 | Yes | - | - | - | - | - | - | - |
| 19 | - | - | - | Thoracic wall reconstruction with net | AI | 75mg/m²  9g/m² | 2 -> Re-Staging -> 6 | 3 weeks |
| 20 | - | - | - | Thoracic wall reconstruction depending on response to RT | - | - | - | - |
| 21 | Yes | - | - | - | Trabectedin or AI | 1.5mg/m² or  75mg/m² d1  2.5g/m² d1-4 | 2 -> Re-Staging | 3 weeks |
| ^*^Center recommends surgery, details on surgery are not available.  **Abbreviations/symbols:** - = Indicates that the center did not select this option. AI = Doxorubicin and Ifosfamide; CTX = Chemotherapy; d= day; Doxo= Doxorubicin; DTIC= Dacarbazine; Epi = Epirubicin; Ifo= Ifosfamide; N/A = Information not available; RT = Radiotherapy | | | | | | | | |

|  | **Case 2: Localized myxoid liposarcoma (G3) in the right shoulder blade** | | | | | | | |
| --- | --- | --- | --- | --- | --- | --- | --- | --- |
|  | Radiotherapy (RT) | | | | Deep regional hyperthermia | | | |
| Center | Type of radiation | Dosage per fraction, fractions per day | Total dosage | Technique | Concurrent with  CTX or RT | No. of sessions per time -> total no. of sessions | Duration per session [min] | Target temperature [°C] |
| 1 | - | - | - | - | - | - | - | - |
| 2 | Photon | 2 Gy, once daily | 50 Gy | VMAT | - | - | - | - |
| 3 | Photon | 2 Gy, once daily | 40 Gy | IMRT | - | - | - | - |
| 4 | Photon | 2 Gy, once daily | 50 Gy | IMRT | - | - | - | - |
| 5 | Photon | 2 Gy, once daily | 50 Gy | IMRT | CTX and RT | 1/week (with CTX)  3/week (with RT)  -> 14 | 60 | 42-44 |
| 6 | Photon | 2 Gy, once daily | 60-66 Gy | IMRT/VMAT/IGRT | CTX | 2/week -> 12 | 90 | 43°C |
| 7 | Photon | 2 Gy, once daily | 50 Gy | IMRT/VMAT/IGRT | - | - | - | - |
| 8 | Photon | 2 Gy, once daily | 50 Gy | 3D-CRT/VMAT | - | - | - | - |
| 9 | Photon | 2 Gy, once daily | 50 Gy | IMRT | - | - | - | - |
| 10 | Photon | 2 Gy, once daily | 36 Gy | IMRT/IGRT | - | - | - | - |
| 11 | Photon | 1.8 Gy, once daily | 50.4 Gy | IMRT/IGRT | - | - | - | - |
| 12 | Photon | 1.8-2 Gy, once daily | 36-50.4 Gy | IMRT | - | - | - | - |
| 13 | Photon | 1.8-2 Gy, once daily | 50-50.4 Gy | IMRT/VMAT | - | - | - | - |
| 14 | Photon | N/A | N/A | N/A | - | - | - | - |
| 15 | Photon | 2 Gy, once daily | 50 Gy | IMRT | - | - | - | - |
| 16 | Photon or proton | 2 Gy, once daily | 36-50 Gy | IMRT/VMAT | CTX | N/A | N/A | N/A |
| 17 | Photon | 2 Gy, once daily | 50 Gy | VMAT | CTX | 2/cycle -> 16 | 90 | 41 |
| 18 | Photon | 1.8-2 Gy, once daily | 50-50.4 Gy | IMRT | - | - | - | - |
| 19 | Photon | 2 Gy, once daily | 50 Gy | IMRT/IGRT | CTX | 2/week -> 12 | N/A | N/A |
| 20 | Photon | 2 Gy, once daily | 36 Gy | 3D-CRT/VMAT/IGRT | - | - | - | - |
| 21 | Photon | 2 Gy, once daily | 50 Gy | IMRT/VMAT/IGRT | Details N/A* | Details N/A* | Details N/A* | Details N/A* |
| ^*^Center recommends regional hyperthermia, details on hyperthermia are not available.  **Abbreviations/symbols:** - = Indicates that the center did not select this option. 3D-CRT = Three-dimensional conformal radiation therapy; CTX = Chemotherapy; d= day, IGRT = Image-guided radiation therapy; IMRT = Intensity modulated radiotherapy; N/A = Information not available; RT = Radiotherapy; SGRT = Surface Guided Radiation Therapy; VMAT = Volumetric Modulated Arc Therapy | | | | | | | | |

|  | **Case 3: Locally recurrent dedifferentiated liposarcoma (G2) in the right retroperitoneum** | | | | |
| --- | --- | --- | --- | --- | --- |
|  | **Surgery** | **Chemotherapy (CTX)** | | | |
| Center | Multivisceral compartment-oriented resection including the following organs | Agent | Dosage | No. of cycles | Duration of each cycle |
| 1 | - | AI | 75mg/m²  9g/m² | 6 | 3 weeks |
| 2 | Right ureter only, possibly colon, right kidney | - | - | - | - |
| 3 | Right hemicolon, right kidney | - | - | - | - |
| 4 | Right hemicolon, right kidney | AI | 60mg/m², 3x3g | 3 -> Re-Staging -> 3 | 3 weeks |
| 5 | Details N/A* | AI | 60mg/m², 9g/m² | 4 | 3 weeks |
| 6 | Right hemicolon, right kidney possibly with autotransplantation | AI | 60mg/m² d1, 3g/m² d1-3 | 3 -> Re-Staging -> 3 | 3 weeks |
| 7 | Details N/A* | AI | 75mg/m² d1,  1.5g d1-4 | 2 ->  Re-Staging -> 2 | 3 weeks |
| 8 | Details N/A* | - | - | - | - |
| 9 | Right hemicolon, possibly right kidney | - | - | - | - |
| 10 | Right hemicolon, right kidney | - | - | - | - |
| 11 | Right hemicolon, right kidney, vena cava, aorta, pancreas | - | - | - | - |
| 12 | Right hemicolon, right kidney, subadventitial vessel dissection | - | - | - | - |
| 13 | Right hemicolon, right kidney, pancreas | - | - | - | - |
| 14 | Right kidney, pancreas | AI | 30mg/m² d1,2,  2g/m² d1-3 | 6 | N/A |
| 15 | Depending on re-staging after RT | - | - | - | - |
| 16 | Right hemicolon, right kidney | AI | 75mg/m² d1,  2500mg/m² d1-4 | 6 | N/A |
| 17 | Right hemicolon, right kidney, psoas muscle | AI | 60mg/m², 6g/m² | 8 | N/A |
| 18 | Details N/A* | - | - | - | - |
| 19 | Right hemicolon, right kidney | AI | 75mg/m², 9g/m² | 2 -> Re-Staging -> 6 | 3 weeks |
| 20 | Right hemicolon, possibly right kidney | - | - | - | - |
| 21 | Right hemicolon, right ureter, possibly right kidney, vena cava, replacement of aorta, possibly pancreas | AI | 60mg/m² d1,  1.5g/m² d1-4 | 2 -> Re-Staging | 3 weeks |
| ^*^Center recommends surgery, details on surgery are not available.  **Abbreviations/symbols:** - = Indicates that the center did not select this option. AI = Doxorubicin and Ifosfamide; CTX = Chemotherapy; d= day; Doxo= Doxorubicin; DTIC= Dacarbazine; Epi = Epirubicin; Ifo= Ifosfamide; N/A = Information not available; RT = Radiotherapy | | | | | |

|  | **Case 3: Locally recurrent dedifferentiated liposarcoma (G2) in the right retroperitoneum** | | | | | | | |
| --- | --- | --- | --- | --- | --- | --- | --- | --- |
|  | Radiotherapy (RT) | | | | Deep regional hyperthermia | | | |
| Center | Type of radiation | Dosage per fraction, fractions per day | Total dosage | Technique | Concurrent with  CTX or RT | No. of sessions per time -> total no. of sessions | Duration per session [min] | Target temperature [°C] |
| 1 | - | - | - | - | - | - | - | - |
| 2 | - | - | - | - | - | - | - | - |
| 3 | Details N/A* | Details N/A* | Details N/A* | Details N/A* | - | - | - | - |
| 4 | - | - | - | - | - | - | - | - |
| 5 | Photon | 1.8 Gy | 45 Gy | IMRT with 4D-CT planning | CTX and RT | 1/week (with CTX)  3/week (with RT)  -> 14 | 60 | 42-44 |
| 6 | - | - | - | - | CTX | 2/week -> 12 | 90 | 43°C |
| 7 | - | - | - | - | - | - | - | - |
| 8 | Photon | 2 Gy, once daily | 50 Gy | VMAT | - | - | - | - |
| 9 | Proton or carbon ion followed by IORT, IORT radiation type N/A | 3 Gy, once daily, IORT dose N/A | 39 Gy (RBE) + IORT dose | Active raster scan technique | - | - | - | - |
| 10 | Photon | 1.8 Gy, once daily | 50.4 Gy | IMRT/IGRT | - | - | - | - |
| 11 | Photon | 1.8 Gy, once daily | 50.4 Gy | IMRT/IGRT | - | - | - | - |
| 12 | Photon | 1.8/2.0 Gy SIB, once daily | 45/50 Gy | IMRT/IGRT | - | - | - | - |
| 13 | - | - | - | - | - | - | - | - |
| 14 | Photon | N/A | N/A | N/A | - | - | - | - |
| 15 | Photon | 2 Gy, once daily | 50 Gy | IMRT | - | - | - | - |
| 16 | - | - | - | - | - | - | - | - |
| 17 | Photon | 1.8 Gy | 50.4 Gy | VMAT | CTX | 2/cycle -> 16 | 90 | 41 |
| 18 | Details N/A* | Details N/A* | Details N/A* | Details N/A* | - | - | - | - |
| 19 | Photon | 2 Gy, once daily | 50 Gy | IMRT/IGRT | CTX | N/A | N/A | N/A |
| 20 | - | - | - | - | - | - | - | - |
| 21 | Details N/A* | Details N/A* | Details N/A* | Details N/A* | - | - | - | - |
| ^*^Center recommends radiotherapy, details on radiotherapy are not available.  **Abbreviations/symbols:** - = Indicates that the center did not select this option. 4D-CT = Four-dimensional computed tomography; CTX = Chemotherapy; d= day, IGRT = Image-guided radiation therapy; IMRT = Intensity modulated radiotherapy; IORT = intraoperative radiotherapy; N/A = Information not available; RBE= Relative biological effectiveness; RT = Radiotherapy; SIB = Simultaneous Integrated Boost; SGRT = Surface Guided Radiation Therapy; VMAT = Volumetric Modulated Arc Therapy | | | | | | | | |

|  | **Case 4: Well-differentiated liposarcoma (G1) in the right retroperitoneum** | | | | |
| --- | --- | --- | --- | --- | --- |
|  | **Surgery** | **Chemotherapy (CTX)** | | | |
| Center | Surgical approach | Agent | Dosage | No. of cycles | Duration of each cycle |
| 1 | Repeat biopsy only | - | - | - | - |
| 2 | Multivisceral compartment-oriented resection:  Right hemicolon, right kidney, pancreas, partial duodenum | - | - | - | - |
| 3 | Multivisceral compartment-oriented resection: right hemicolon, right kidney | - | - | - | - |
| 4 | Multivisceral compartment-oriented resection: right hemicolon, right kidney | - | - | - | - |
| 5 | Organ-preserving surgery: right hemicolon, right lower pole of kidney | - | - | - | - |
| 6 | Multivisceral compartment-oriented resection: Right kidney | - | - | - | - |
| 7 | Details N/A* | - | - | - | - |
| 8 | Organ-preserving surgery | - | - | - | - |
| 9 | Multivisceral compartment-oriented resection: right hemicolon, right kidney | - | - | - | - |
| 10 | Multivisceral compartment-oriented resection: right hemicolon, right kidney | - | - | - | - |
| 11 | Multivisceral compartment-oriented resection: right hemicolon, right kidney,  vena cava, aorta, pancreas | - | - | - | - |
| 12 | Multivisceral compartment-oriented resection: right hemicolon, right kidney,  duodenum, retroperitoneal wall, subadventitial vessel dissection | - | - | - | - |
| 13 | Multivisceral compartment-oriented resection: right hemicolon, right kidney, pancreas | - | - | - | - |
| 14 | Multivisceral compartment-oriented resection: right hemicolon, right kidney | - | - | - | - |
| 15 | Multivisceral compartment-oriented resection: right kidney | - | - | - | - |
| 16 | Multivisceral compartment-oriented resection: right hemicolon, right kidney | - | - | - | - |
| 17 | Multivisceral compartment-oriented resection: right hemicolon, right kidney | - | - | - | - |
| 18 | Details N/A* | - | - | - | - |
| 19 | Multivisceral compartment-oriented resection: right kidney | - | - | - | - |
| 20 | Multivisceral compartment-oriented resection: right hemicolon, right kidney, possibly duodenum and vena cava, depending on intraoperative findings | - | - | - | - |
| 21 | Organ-preserving surgery | - | - | - | - |
| ^*^Center recommends surgery, details on surgery are not available.  **Abbreviations/symbols:** - = Indicates that the center did not select this option. AI = Doxorubicin and Ifosfamide; CTX = Chemotherapy; d= day; Doxo= Doxorubicin; DTIC= Dacarbazine; Epi = Epirubicin; Ifo= Ifosfamide; N/A = Information not available; RT = Radiotherapy | | | | | |

|  | **Case 4: Well-differentiated liposarcoma (G1) in the right retroperitoneum** | | | | | | | |
| --- | --- | --- | --- | --- | --- | --- | --- | --- |
|  | Radiotherapy (RT) | | | | Deep regional hyperthermia | | | |
| Center | Type of radiation | Dosage per fraction, fractions per day | Total dosage | Technique | Concurrent with  CTX or RT | No. of sessions per time -> total no. of sessions | Duration per session [min] | Target temperature [°C] |
| 1 | - | - | - | - | - | - | - | - |
| 2 | - | - | - | - | - | - | - | - |
| 3 | - | - | - | - | - | - | - | - |
| 4 | - | - | - | - | - | - | - | - |
| 5 | - | - | - | - | - | - | - | - |
| 6 | - | - | - | - | - | - | - | - |
| 7 | Photon | 1.8 Gy, once daily | 50.4 Gy | IMRT/VMAT/IGRT | - | - | - | - |
| 8 | - | - | - | - | - | - | - | - |
| 9 | Proton or carbon ion followed by IORT, IORT radiation type N/A | 3 Gy, once daily, IORT dose N/A | 39 Gy (RBE) + IORT dose | Active raster scan technique | - | - | - | - |
| 10 | - | - | - | - | - | - | - | - |
| 11 | Photon | 1.8 Gy, once daily | 50.4 Gy | IMRT/IGRT | - | - | - | - |
| 12 | Photon | 1.8/2.0 Gy SIB, once daily | 45/50 Gy | IMRT/IGRT | - | - | - | - |
| 13 | - | - | - | - | - | - | - | - |
| 14 | Details N/A* | Details N/A* | Details N/A* | Details N/A* | - | - | - | - |
| 15 | Photon | 2 Gy, once daily | 50 Gy | IMRT | - | - | - | - |
| 16 | - | - | - | - | - | - | - | - |
| 17 | Photon | 1.8 Gy | 50.4 Gy | VMAT | - | - | - | - |
| 18 | Details N/A* | Details N/A* | Details N/A* | Details N/A* | - | - | - | - |
| 19 | Photon | 2 Gy, once daily | 50 Gy | N/A | - | - | - | - |
| 20 | - | - | - | - | - | - | - | - |
| 21 | - | - | - | - | - | - | - | - |
| ^*^Center recommends radiotherapy, details on radiotherapy are not available.  **Abbreviations/symbols:** - = Indicates that the center did not select this option. 4D-CT = Four-dimensional computed tomography; CTX = Chemotherapy; d= day, IGRT = Image-guided radiation therapy; IMRT = Intensity modulated radiotherapy; IORT = intraoperative radiotherapy; N/A = Information not available; RBE= Relative biological effectiveness; RT = Radiotherapy; SIB = Simultaneous integrated boost; SGRT = Surface Guided Radiation Therapy; VMAT = Volumetric modulated arc therapy | | | | | | | | |

|  | **Case 5: Localized pleomorphic rhabdomyosarcoma (G3) in the right popliteal fossa** | | | | |
| --- | --- | --- | --- | --- | --- |
|  | **Surgery** | **Chemotherapy (CTX)** | | | |
| Center | Surgical approach | Agent | Dosage | No. of cycles | Duration of each cycle |
| 1 | Details N/A* | AI | 70mg/m²  8g/m² | 6 | 3 weeks |
| 2 | Amputation | - | - | - | - |
| 3 | Wide resection with replacement of popliteal artery | AI | 60mg/m² d1, 3g/m² d1-3 | AI: 4  Ifo (+ RT): 2 | 3 weeks |
| 4 | Wide resection with primary wound closure | Details N/A* | Details N/A* | Details N/A* | Details N/A* |
| 5 | Details N/A* | Ifo | 3g/m² d1,2 | 2 | 3 weeks |
| 6 | Wide resection with plastic reconstruction | AI | Details N/A* | Details N/A* | Details N/A* |
| 7 | Details N/A* | AI | 75mg/m² d1, 1.5g d1-4 | 2 -> Re-Staging -> 2 | 3 weeks |
| 8 | Details N/A* | - | - | - | - |
| 9 | Wide resection with replacement of popliteal artery | - | - | - | - |
| 10 | Wide resection of popliteal vessels; artery replaced, vein not replaced | AI | 60mg/m²  6g/m² | 4-6 | 3 weeks |
| 11 | Wide resection with replacement of popliteal artery | AI | 75mg/m²  9g/m² | 5 | 3 weeks |
| 12 | ILP followed by wide resection with replacement of popliteal vessels | AI | 60mg/m² d1  1.8g/m² d1-5 | 4 | 4 weeks |
| 13 | Wide resection with replacement of popliteal vessels | AI | 60mg/m² d1 2.5g/m² d1-4 | 5 | 3 weeks |
| 14 | ILP followed by wide resection with replacement of popliteal vessels | AI | 30mg/m² d1,2  2g/m² d1-3 | 6 | N/A |
| 15 | Reference pathological examination. Re-biopsy, if necessary | - | - | - | - |
| 16 | Wide resection with replacement of popliteal artery | Details N/A* | Details N/A* | Details N/A* | Details N/A* |
| 17 | Depending on tumor response to neoadjuvant treatments | AI | 60mg/m²  6g/m² | 8 | N/A |
| 18 | ILP only | - | - | - | - |
| 19 | ILP followed by wide resection with replacement of popliteal vessels | AI | 75mg/m²  9g/m² | 2 -> Re-Staging -> 6 | 3 weeks |
| 20 | Wide resection with marginal resection at tibial nerve, replacement of popliteal vessels, possibly plastic reconstruction | - | - | - | - |
| 21 | Wide resection with replacement of femoral/ popliteal artery, veins not routinely replaced | Vincristine, Dactinomycin, Cyclophosphamide | 1.5mg/m², 0.045mg/kg, 1200g/m² | 8 | 3 weeks |
| ^*^Center recommends treatment modality, details on treatment modality are not available.  **Abbreviations/symbols:** - = Indicates that the center did not select this option. AI = Doxorubicin and Ifosfamide; CTX = Chemotherapy; d= day; Doxo= Doxorubicin; DTIC= Dacarbazine; Epi = Epirubicin; Ifo= Ifosfamide; ILP = Isolated limb perfusion N/A = Information not available; RT = Radiotherapy | | | | | |

|  | **Case 5: Localized pleomorphic rhabdomyosarcoma (G3) in the right popliteal fossa** | | | | | | | |
| --- | --- | --- | --- | --- | --- | --- | --- | --- |
|  | Radiotherapy (RT) | | | | Deep regional hyperthermia | | | |
| Center | Type of radiation | Dosage per fraction, fractions per day | Total dosage | Technique | Concurrent with  CTX or RT | No. of sessions per time -> total no. of sessions | Duration per session [min] | Target temperature [°C] |
| 1 | - | - | - | - | CTX | 2/cycle -> 12 | 90 | 42 |
| 2 | - | - | - | - | - | - | - | - |
| 3 | Photon | 2 Gy, once daily | 50 Gy | IMRT | - | - | - | - |
| 4 | Photon | 2 Gy, once daily | N/A | IMRT | - | - | - | - |
| 5 | Photon | 2 Gy, once daily | 50 Gy | IMRT | RT | 2/week -> 10 | 60 | 42-44 |
| 6 | - | - | - | - | - | - | - | - |
| 7 | Details N/A* | Details N/A* | Details N/A* | Details N/A* | - | - | - | - |
| 8 | - | - | - | - | - | - | - | - |
| 9 | Photon | 2 Gy, once daily | 50 Gy | IMRT | - | - | - | - |
| 10 | Details N/A* | Details N/A* | Details N/A* | Details N/A* | CTX | 2/cycle -> 12 | N/A | N/A |
| 11 | Details N/A* | Details N/A* | Details N/A* | Details N/A* | CTX | N/A | N/A | N/A |
| 12 | Details N/A* | Details N/A* | Details N/A* | Details N/A* | - | - | - | - |
| 13 | Photon | 1.8-2 Gy, once daily | 50-50.4 Gy | IMRT/VMAT | - | - | - | - |
| 14 | Photon | N/A | N/A | N/A | - | - | - | - |
| 15 | - | - | - | - | - | - | - | - |
| 16 | - | - | - | - | - | - | - | - |
| 17 | Photon | 2 Gy, once daily | 50 Gy | VMAT | CTX | 2/cycle -> 16 | 90 | 41 |
| 18 | - | - | - | - | - | - | - | - |
| 19 | Photon | 2 Gy, once daily | 50 Gy | IMRT/IGRT | CTX | 2/week -> 12 | N/A | N/A |
| 20 | Photon | 2 Gy, once daily | 66-70 Gy | VMAT/IGRT/SGRT | - | - | - | - |
| 21 | Photon | 2 Gy, once daily | 50 Gy | IMRT/VMAT/IGRT | Details N/A* | Details N/A* | Details N/A* | Details N/A* |
| ^*^Center recommends treatment modality, details on treatment modality are not available. **Abbreviations/symbols:** - = Indicates that the center did not select this option. CTX = Chemotherapy; d= day, IGRT = Image-guided radiation therapy; IMRT = Intensity modulated radiotherapy; N/A = Information not available; RT = Radiotherapy; SGRT = Surface Guided Radiation Therapy; VMAT = Volumetric Modulated Arc Therapy | | | | | | | | |
